# Commentary on: Recollection reduces unitised familiarity effect

**DOI:** 10.3389/fpsyg.2015.00757

**Published:** 2015-06-02

**Authors:** Roni Tibon, Richard Henson

**Affiliations:** Cognition and Brain Sciences Unit, Medical Research CouncilCambridge, UK

**Keywords:** familiarity, recollection, unitization, dual-process model, episodic memory

The dual-process theory of recognition memory posits that recognition is supported by two separable processes: familiarity and recollection. Familiarity is the feeling of previously encountering something, without retrieval of contextual information about that encounter, whereas recollection refers to additional retrieval of contextual details (Yonelinas, 2002). While it is generally agreed that recognition of a single item can be supported by both processes, memory for novel associations between items is usually thought to require recollection (e.g., Yonelinas, 1997; Donaldson and Rugg, 1998; Hockley and Consoli, 1999). Nonetheless, one situation when memory for an association between items might be supported by familiarity is when the items are bound together as a single unit; so-called “unitization” (e.g., Yonelinas et al., 1999; Rhodes and Donaldson, 2008; Jäger and Mecklinger, 2009; Diana et al., 2011; Tibon et al., 2014a,b).

Shao et al. (2015) recently reported interesting differences between two tasks that have been used to investigate the effects of unitization on associative recognition. In the first, *compound task* (Experiments 1 and 2), participants' memory for initially-unrelated stimuli was tested after two types of encoding. In the definition condition, the words were given a definition that enabled a new, unitized concept. This condition was compared against the sentence condition, where the words were presented as separate components of a sentence. In the second, *imagery task* (Experiment 3), the same types of words were used, but unitization was tested by comparing interactive-imagery condition (“create an image of the items interacting together”) with an item-imagery condition (“create a separate image for each item”).

Shao et al.'s driving hypothesis was that the imagery task engages more recollection than the compound task, due to the flexibility afforded by adopting self-generated, elaborative encoding strategies. They further suggested that this increased recollection reduces the contribution of familiarity to the associative recognition task. They tested this hypothesis by combining both types of task with the Remember/Know procedure. In Experiment 1, they replicated the unitization advantage in the compound task. In Experiment 2 they found that this advantage was associated with increased familiarity. However, when using the imagery task in Experiment 3, they found that interactive imagery, which was supposed to encourage unitization, produced increased recollection and reduced familiarity. The authors claimed that this supports their hypothesis that when the memory trace is easily recollected (e.g., following interactive-imagery), the need for familiarity assessment is redundant, and so the effect of unitization on familiarity disappears. Although intriguing, we would like to offer alternative perspectives on both methodological and theoretical aspects of their report, which lead to the opposite conclusion.

Our methodological concern relates to the procedure used in the imagery task. The finding of reduced familiarity in the interactive condition is inconsistent with a previous study by Rhodes and Donaldson (2008). One difference between the two studies is that, in the item-imagery condition, Shao et al. instructed participants to indicate which one of the two mental images was clearer. These instructions would seem to encourage some interaction between images, which would in turn foster unitization. Thus it is possible that unitization occurred in *both* of Shao et al.'s encoding conditions (the authors do acknowledge this, but for a different reason). If so, the reason why familiarity estimates were higher in the item-imagery than interactive-imagery condition would be because Shao et al. used a shorter study, and repeated each item twice, in the item-imagery condition. Though this was supposed to match overall memory performance across the two conditions, a possible consequence is that familiarity was actually stronger in the item-imagery condition.

This leads us to our theoretical concern, regarding interactions between recollection and familiarity. The dominant view of recollection and familiarity is that they are independent (Yonelinas, 2002). Nonetheless, Shao et al. claim that the two processes can sometimes interact; specifically, that increasing recollection can decrease familiarity. However, it is equally possible that when familiarity is readily available, participants do not need to engage in effortful recollection. This opposite type of interaction is in line with other theoretical characteristics of recollection and familiarity. In particular, familiarity is often claimed to be automatic, and occurs faster than recollection (Yonelinas, 2002; Diana et al., 2006; though see Moscovitch, 2008). Indeed, ERP studies reveal ample evidence of earlier onset of the putative “FN400” correlate of familiarity than the “LPC” correlate of recollection (e.g., Mecklinger, 2000; Rugg and Curran, 2007; Wilding and Ranganath, 2011). But perhaps the most compelling evidence for our proposal comes from the ERP study of Bader et al. (2010). These authors used the compound task, and found the FN400 effect for unitized word-pairs, whereas the LPC was only elicited by non-unitized word-pairs. Given that the familiarity-related modulation occurred earlier, these authors claimed, like us, that familiarity can sometimes be sufficiently diagnostic to support associative recognition, obviating the need for additional recollection.

These considerations have wider implications, because evidence that recollection and familiarity interact would question the common assumption that these processes operate independently. Note that recollection and familiarity can show parallel or even opposing effects of another variable without interacting, so conclusive evidence for such interaction is difficult to obtain. Nonetheless, if we assume, like Shao et al suggest, that changes in recollection can sometimes cause changes in familiarity, one should not find an experimental variable (like unitization) that affects familiarity without also affecting recollection (though it is possible for recollection to change without familiarity changing). According to our alternative, however, one should not find a variable that affects recollection without also affecting familiarity (though it is possible for familiarity to change without recollection changing). There is some evidence against both of these possibilities (Figure 1). Again, both of these patterns can be explained if recollection and familiarity are independent. Therefore, we believe the consideration of data like Shao et al's raises important questions about whether, and if so when and how, familiarity and recollection interact to determine people's recognition judgments.

**Figure 1:**
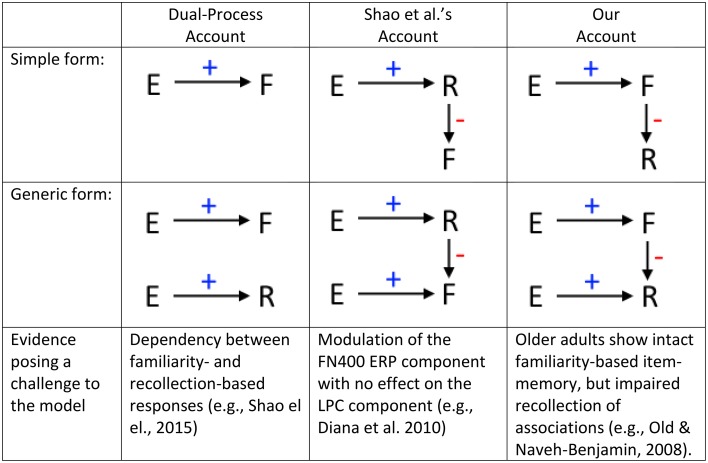
**Formulization of plausible models according to the dual-process account (e.g., Yonelinas, 2002), Shao et al. (2015)'s account, and our account**. The simple form of each model (top row) represents a case in which a variable (E, e.g., unitization) affects one recognition process (either familiarity, F, or recollection, R), which in turn affects the other. In the generic form (bottom row), E affects both recognition processes. Using this depiction, one can see why a change in familiarity but no change in recollection would be problematic for Shao's model (which assumes the route to diminished F is via increased R). Similarly, a change in recollection but no change in familiarity would be problematic for our model (which assumes the route to diminished R is via increased F).

## Conflict of interest statement

The authors declare that the research was conducted in the absence of any commercial or financial relationships that could be construed as a potential conflict of interest.
